# HDAC7 Is a Repressor of Myeloid Genes Whose Downregulation Is Required for Transdifferentiation of Pre-B Cells into Macrophages

**DOI:** 10.1371/journal.pgen.1003503

**Published:** 2013-05-16

**Authors:** Bruna Barneda-Zahonero, Lidia Román-González, Olga Collazo, Haleh Rafati, Abul B. M. M. K. Islam, Lars H. Bussmann, Alessandro di Tullio, Luisa De Andres, Thomas Graf, Núria López-Bigas, Tokameh Mahmoudi, Maribel Parra

**Affiliations:** 1 Cancer Epigenetics and Biology Program (PEBC), Bellvitge Biomedical Research Institute (IDIBELL), Barcelona, Spain; 2 Erasmus University Medical Center, Department of Biochemistry, Rotterdam, The Netherlands; 3 Research Unit on Biomedical Informatics, Department of Experimental and Health Sciences, Universitat Pompeu Fabra, Barcelona, Spain; 4 Center for Genomic Regulation, Barcelona, Spain; 5 Institució Catalana de Recerca i Estudis Avançats (ICREA), Barcelona, Spain; Cincinnati Children's Hospital Medical Center, United States of America

## Abstract

B lymphopoiesis is the result of several cell-commitment, lineage-choice, and differentiation processes. Every differentiation step is characterized by the activation of a new, lineage-specific, genetic program and the extinction of the previous one. To date, the central role of specific transcription factors in positively regulating these distinct differentiation processes to acquire a B cell–specific genetic program is well established. However, the existence of specific transcriptional repressors responsible for the silencing of lineage inappropriate genes remains elusive. Here we addressed the molecular mechanism behind repression of non-lymphoid genes in B cells. We report that the histone deacetylase HDAC7 was highly expressed in pre-B cells but dramatically down-regulated during cellular lineage conversion to macrophages. Microarray analysis demonstrated that HDAC7 re-expression interfered with the acquisition of the gene transcriptional program characteristic of macrophages during cell transdifferentiation; the presence of HDAC7 blocked the induction of key genes for macrophage function, such as immune, inflammatory, and defense response, cellular response to infections, positive regulation of cytokines production, and phagocytosis. Moreover, re-introduction of HDAC7 suppressed crucial functions of macrophages, such as the ability to phagocytose bacteria and to respond to endotoxin by expressing major pro-inflammatory cytokines. To gain insight into the molecular mechanisms mediating HDAC7 repression in pre-B cells, we undertook co-immunoprecipitation and chromatin immunoprecipitation experimental approaches. We found that HDAC7 specifically interacted with the transcription factor MEF2C in pre-B cells and was recruited to MEF2 binding sites located at the promoters of genes critical for macrophage function. Thus, in B cells HDAC7 is a transcriptional repressor of undesirable genes. Our findings uncover a novel role for HDAC7 in maintaining the identity of a particular cell type by silencing lineage-inappropriate genes.

## Author Summary

Through the hematopoietic system, all the distinct mature blood cell types are generated, thereby constituting one of the best-studied paradigms for cell lineage commitment and differentiation in biology. B lymphocytes are generated through several cell-commitment, lineage-choice, and differentiation processes. To date, the central role of lineage-specific transcription factors in positively regulating these distinct developmental steps is well established. However, in the absence of proper transcriptional repression, an “adolescent cell” will never be able to reach its “adulthood identity,” having a potential impact in the development of hematological malignancies. In this article, we examined the molecular mechanism responsible for the gene silencing of lineage undesirable genes in B cell precursors and uncovered the role played in this process by the histone deacetylase HDAC7. We show that HDAC7 is expressed in B cell precursors where it interacts with the transcription factor MEF2C and is recruited to the promoters of non-B cell genes. While HDAC7 is down-regulated during the lineage conversion of pre-B cells into macrophages, re-expression of HDAC7 interferes with both the acquisition of the myeloid gene transcriptional program and macrophage-specific cell functions. We therefore have identified a novel lineage-specific transcriptional repressor in the hematopoietic system.

## Introduction

The generation of B cells is the result of several cellular transitions that take place in a stepwise manner and comprise cell lineage choices, cell commitment and differentiation. Every differentiation step leads to the activation of specific genes characteristic of the new cellular stage. This is achieved by the action of well defined networks of transcription factors specific to each particular cellular state [1], [2]. In the bone marrow, lymphocyte development begins at the lymphoid-primed multipotent progenitor (LMPPs) stage. LMPPs become common lymphoid progenitors (CLPs), which have the potential to differentiate into B and T lymphocytes, as well as natural killer (NK) cells [3]. The transcription factors IKAROS, PU.1 and MEF2C are critical for the cellular commitment of LMPPs to the lymphoid lineage [3]–[5]. Later, the transcription factors E2A, EBF and FOXO-1 are required for the early specification of CLPs into pro-B cells, whereas PAX5 is required to maintain B cell identity along differentiation into mature B cells [6]–[11]. However, there is an increasing body of evidence indicating that the repression of lineage inappropriate genes is a pivotal mechanism to properly acquire a particular cellular state during B lymphopoiesis. For example, PAX5 not only induces the expression of a B-cell specific genetic program, it also suppresses inappropriate genes of alternative lineages, thereby ensuring its role in maintaining B cell identity and differentiation [12]–[14]. Recently, it has been reported that the transcription factor MEF2C, by activating lymphoid specific genes and repressing myeloid genes, is involved in the cellular choice towards the lymphoid lineage [5]. These studies suggest that B cell transcription factors must also recruit transcriptional co-repressors to silence undesirable genes. To date, very little is known on the role of transcriptional repressors during B lymphopoiesis.

Histone deacetylases (HDACs) have emerged as crucial transcriptional co-repressors in highly diverse physiological systems. To date, 18 human HDACs have been identified and grouped into four classes. Class I HDACs (HDAC1, 2, 3, and 8), class II HDACs (HDAC4, 5, 6, 7, 9, and 10), class III HDACs, also called sirtuins, (SIRT1, 2, 3, 4, 5, 6, and 7) and class IV HDAC (HDAC11). Class II HDACs are further subdivided into class IIa (HDAC4, 5, 7, 9) and class IIb (HDAC6 and 10) [15], [16]. Unlike other HDACs, Class IIa HDACs have three unique features. First, they are expressed in a tissue-specific manner and are involved in development and differentiation processes. They exert their transcriptional repressive function in skeletal, cardiac, and smooth muscle, the bone, the immune system, the vascular system, and the brain among others. Second, they are signal-dependent co-repressors which become phosphorylated at conserved serine residues in the regulatory N-terminal domain leading to their nuclear export. Third, they contain a regulatory N-terminal domain that mediates their interactions with tissue-specific transcription factors such as members of the MEF2 family [15], [16]. This last feature prompted us to ask whether members of the class IIa HDACs subfamily could be lineage-specific transcriptional repressors crucial to maintain B cell identity and biology.

To address this question we have used a cellular transdifferentiation system that we reported recently [17]. This system consists of a pre-B cell line (HAFTL cells) transduced with a retroviral vector for stable expression of a β-estradiol-inducible form of C/EBPα (C10 and C11 cells). After addition of β-estradiol, C10 and C11 cells are converted into functional macrophage-like cells at 100% efficiency within 48–72 hours. The conversion of pre-B cells into macrophages is direct and does not involve overt retro-differentiation through hematopoietic stem and progenitor cells [18]. Unexpectedly, this cellular transdifferentiation process appears to occur in the absence of significant changes in the DNA methylation status of key lymphoid or myeloid genes, but involves changes in histone modification on both types of genes [19]. Since HAFTL cells are a fetal liver cell line immortalized by Ha-ras transformation we, in parallel, also investigated the involvement of class IIa HDACs in normal primary B cell precursors. Here, we report that during the conversion of pre-B cells into macrophages, HDAC7 expression is down-regulated. In pre-B cells HDAC7 specifically interacts with the transcription factor MEF2C and is recruited to putative MEF2 sites located at the promoters of key macrophage genes. Forced re-expression of HDAC7 interferes with the establishment of the gene transcriptional program and functional characteristics of macrophages. Importantly, HDAC7 depletion in pre-B cells results in the de-repression of macrophage genes.

## Results

### HDAC7 is down-regulated during the transdifferentiation of pre-B cells into macrophages

**Figure 1:**
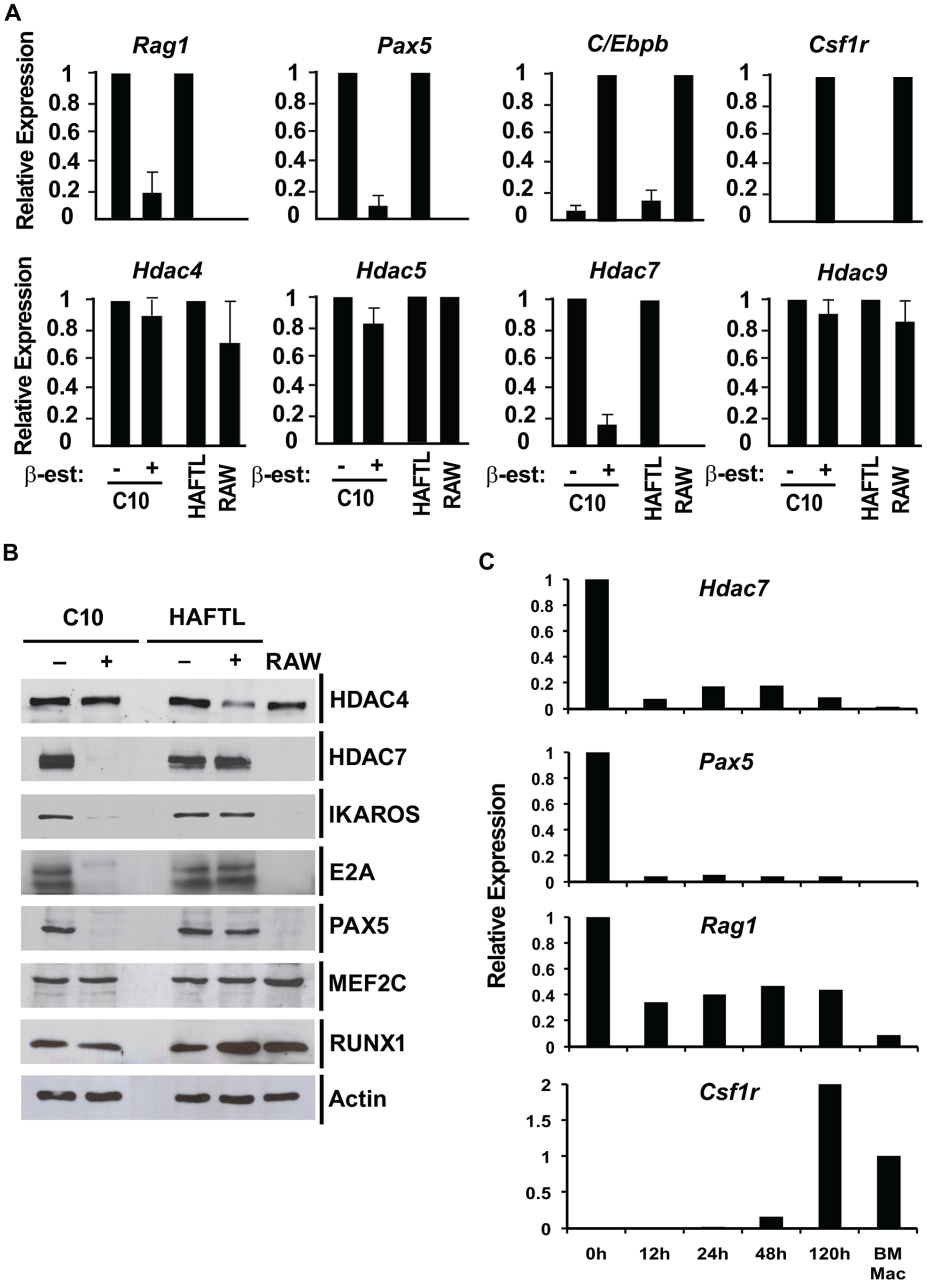
HDAC7 is down-regulated during the transifferentiation of pre-B cells into macrophages. A. RT-qPCR experiments for gene expression changes of class IIa HDACs, B cell and macrophages genes (uninduced cells (−) and β-estradiol induced C10 cells (+) for 72 hours). HAFTL pre-B cells and RAW264.7 cells were used as control. Data are represented as the mean +/− standard error of the mean (SEM) of three independent experiments. B. Western blot of Class IIa HDACs and B cell transcription factors in the same experimental conditions shown in A. C. RT-qPCR experiments for gene expression changes of *Hdac7*, *Pax5*, *Rag1* and *Csf1r* genes (uninduced (0) and β-estradiol induced primary pre-B cells transduced with a retroviral vector for C/EBPα expression for the indicated times. Primary macrophages were used as control.

In order to study the potential role of class IIa HDACs in B lymphocyte biology we first analyzed their expression levels during the cellular transdifferentiation of pre-B cells into macrophages. RT-qPCR and Western blotting experimental approaches showed that HDAC7 expression was dramatically down-regulated during the conversion of C10 cells into macrophages at 72 hours after β-estradiol treatment (Figure 1A and 1B). Notably, no changes in *Hdac4*, *Hdac5* and *Hdac9* expression levels were observed during the cellular reprogramming process. As previously described [17], the B cell genes *Rag1* and *Pax5* became down-regulated, whereas the expression of the myeloid genes *C/Ebpb* and *Csf1r* were up-regulated during cellular reprogramming (Figure 1A). Consistent with our findings, HDAC7 is not present in RAW264.7 macrophages at neither the RNA nor protein levels (Figure 1A and 1B). As expected, the expression of the B cell transcription factors IKAROS, E2A, EBF and PAX5 were down-regulated during the cellular conversion. In contrast, protein levels of the transcription factors MEF2C and RUNX1 did not significantly change during transdifferentiation (Figure 1B). To further confirm our findings we analyzed the kinetics of HDAC7 expression during the cellular transdifferentiation in our previously reported microarray experiments [17]. Strikingly, similar to the B cell specific transcription factor PAX5, HDAC7 expression was down-regulated during cellular reprogramming (Figure S1). Since HAFTL cells are a fetal-derived pre-B cell line transformed by Ha-ras we wondered whether our findings could be extended to normal primary cells. We analyzed HDAC7 expression in both primary B cell precursors and primary macrophages in a recently reported microarray analysis [18]. Importantly, we found that, similar to PAX5, HDAC7 was highly expressed in B cell precursors compared to primary macrophages (Figure 1C and Figure S1). In agreement with our results with the C10 cell line, C/EBPα-mediated conversion of primary pre-B cells into macrophages also resulted in the down-regulation of HDAC7 (Figure 1C and Figure S1). Our data demonstrate that among the different class IIa HDACs, HDAC7 shows a lymphoid-specific expression pattern and suggest that by repressing lineage inappropriate genes in pre-B cells, such as genes characteristic of macrophages, HDAC7 might be crucial in maintaining B cell functions and identity.

### HDAC7 re-expression interferes with the gene transcriptional program of the reprogrammed macrophages

**Figure 2:**
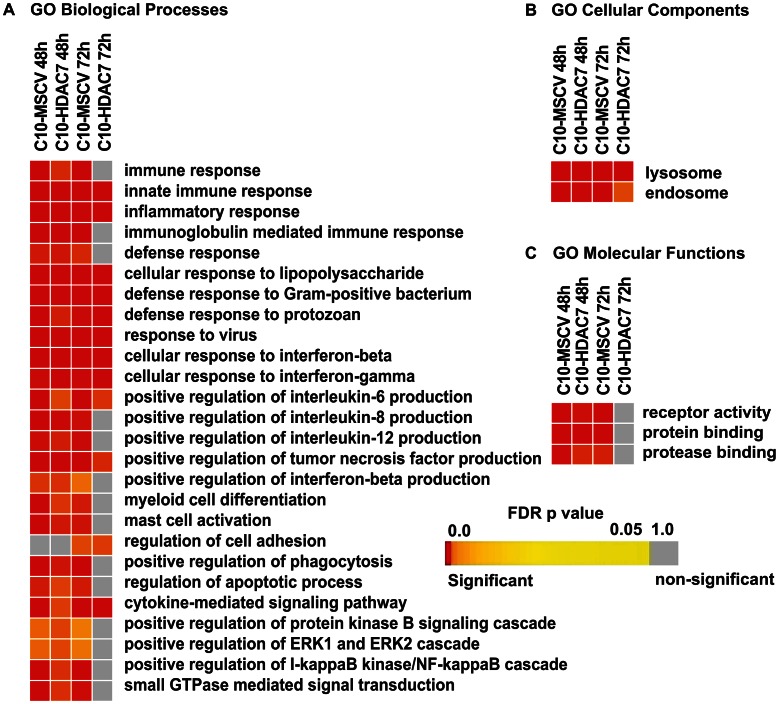
HDAC7 re-expression interferes with the gene transcriptional program of the converted macrophages. Heat-maps showing significantly (corrected p-value <0.05) enriched GO Biological Process (A), Cellular Components (B) and Molecular Functions (C) categories among the up-regulated genes affected by the re-expression of HDAC7 during transdifferentiation of pre-B cells into macrophages. Colours toward red indicate high statistic significance, yellow indicates low statistic significance, and gray indicates no statistical significance. The list of genes for the enrichment analysis in each column is as follows: C10-MSCV-48h includes genes up-regulated after β-estradiol treatment for 48 hours. C10-HDAC7-48h includes genes up-regulated after β-estradiol treatment for 48 hours which are down-regulated in the presence of HDAC7 (HDAC7 re-expression). C10-MSCV-72h includes genes up-regulated after β-estradiol treatment for 72 hours. C10-HDAC7-72h includes genes up-regulated after β-estradiol treatment for 72 hours which are down-regulated after re-expression of HDAC7.

**Figure 3:**
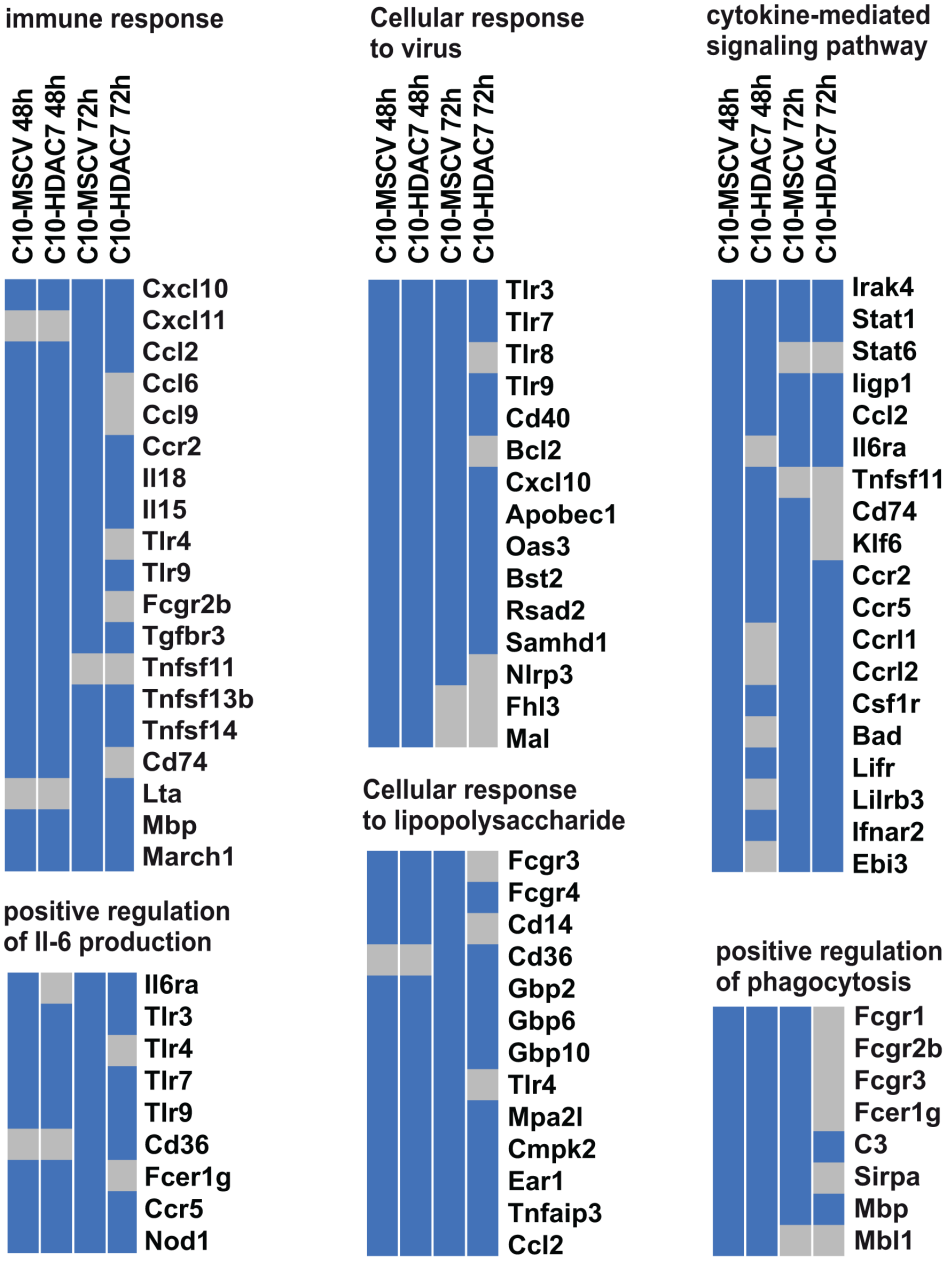
HDAC7 re-expression interferes with the gene transcriptional program of the converted macrophages. Heat-maps showing observed differentially expressed genes for selected enriched GO categories. Blue colour cell indicates positive events while gray colour indicates that the gene was not observed differentially expressed in that experimental condition.

**Figure 4:**
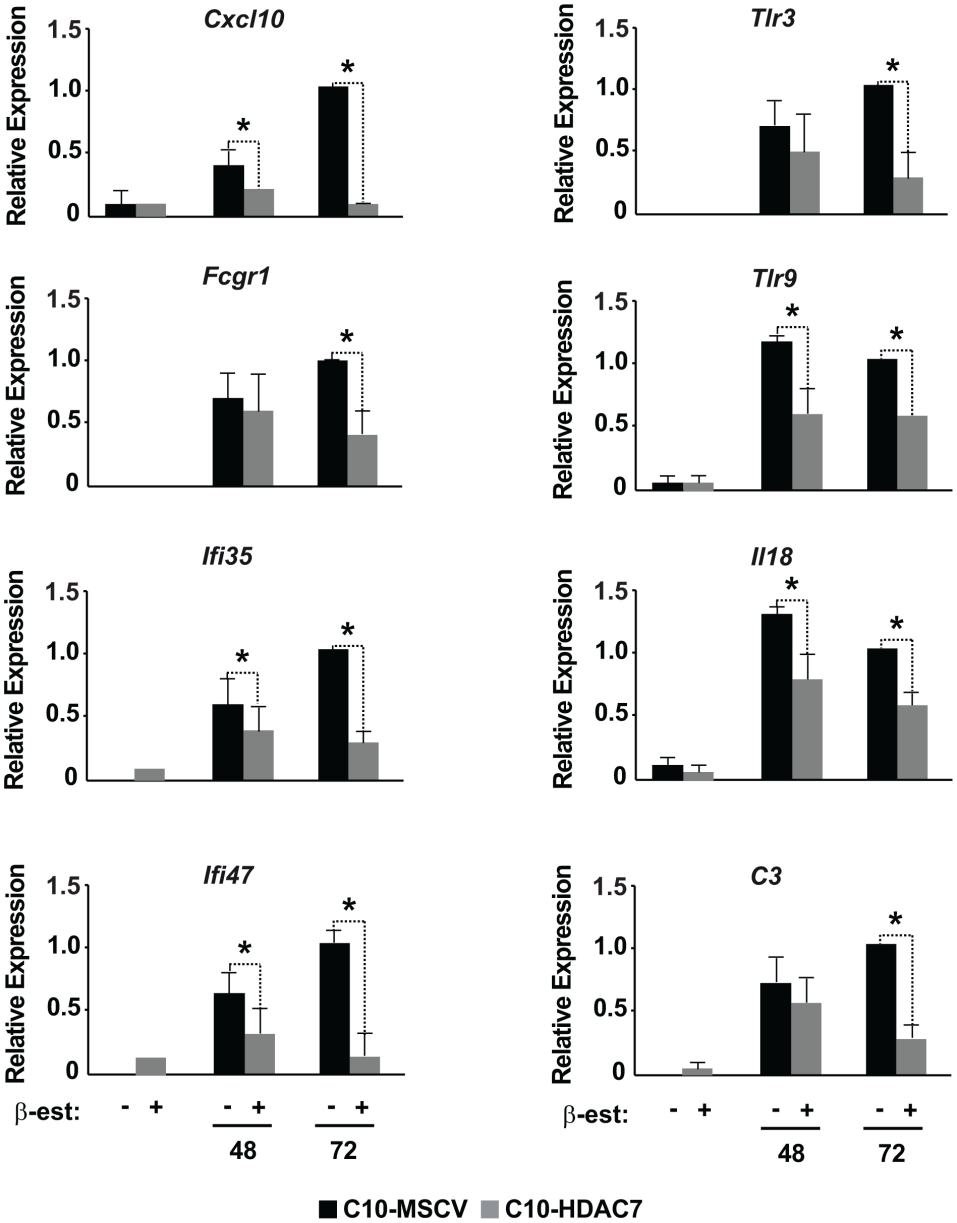
RT–qPCR for microarray validation of selected genes. RT-qPCR experiments for gene expression changes for 8 up-regulated genes, *Cxcl10*, *Fcgr1*, *Ifi35*, *Ifi47*, *Tlr3*, *Tlr9*, *Il18* and *C3* in the absence or in the presence of HDAC7. Data are represented as the mean +/− standard error of the mean (SEM) of three independent experiments. The two-way ANOVA test was used to calculate significant levels between the indicated groups. *P<0.001.

Our observed dramatic down-regulation of HDAC7 upon transdifferentiation of pre-B cells into the macrophage lineage was consistent with its potential role as a transcriptional repressor of macrophage-specific genes in pre-B cells. To test whether the presence of HDAC7 could interfere with the acquisition of a macrophage-specific gene program during transdifferentiation of pre-B cells into macrophages, we performed a gain of function experimental approach followed by genome-wide microarray analysis. We transduced C10 cells with a retroviral vector carrying HDAC7-Flag (C10-HDAC7 cells). As a control, C10 cells were transduced with an empty retroviral vector (C10-MSCV cells). As expected, β-estradiol treatment of C10-MSCV cells resulted in the total down-regulation of endogenous HDAC7 protein levels, while C10-HDAC7 cells expressed the exogenous HDAC7 protein at similar levels as in untreated C10-MSCV cells even at 72 hours after β-estradiol treatment (Figure S2). We then examined the genome-wide effects of HDAC7 re-expression on the gene transcription program of C10 cells induced to transdifferentiate to macrophages. Microarray experiments were conducted in both C10-MSCV and C10-HDAC7 cells un-induced or induced to transdifferentiate for 48 and 72 hours. The addition of β-estradiol to C10-MSCV cells resulted in the up- and down-regulation of 1609 and 1798 genes at 48 hours and of 1531 and 1567 genes at 72 hours after treatment, in agreement with our previous report [17]. Importantly, the exogenous expression of HDAC7 in C10-HDAC7 cells treated with β-estradiol totally or partially abrogated the up-regulation of 988 and 866 genes, after 48 and 72 hours respectively. Earlier analyses showed that the vast majority of the up-regulated genes correspond to key genes for macrophage function, whereas the down-regulated genes are associated with cell cycle processes and with important functions for B cell development and biology [17]. To determine the type of genes whose up-regulation was affected by the presence of HDAC7 we performed a gene set enrichment analysis based on the gene ontology (GO) categories corresponding to Biological Processes, Cellular Components and Molecular Functions, as well as on KEGG pathways. To do so we took advantage of Gitools, a recently developed bioinformatics tool [20]. Gitools allows accessing many available biological databases, performing analysis and visualizing data using interactive heat-maps. Strikingly, the Biological Process enrichment revealed that the set of up-regulated genes affected by HDAC7 belong to GO categories representing key macrophage related functions, such as immune, inflammatory and defense response, cellular response to infections, positive regulation of cytokines production and phagocytosis (Figure 2A). Importantly, we found that the categories enriched in the KEGG pathways analysis correspond to similar biological processes (Figure S3). Gene ontology (GO) analysis corresponding to Cellular Components and Molecular Functions reinforce the above results (Figure 2B and 2C). Among the genes whose up-regulation is impaired by HDAC7 we found several that are involved in phagocytosis (such as *Fcgr1*, *Fcgr2b* and *Fcgr3*), genes related to the immune response (such as the chemokine genes *Cxcl10*, *Cxcl11* and *Ccl2*), Toll-like receptors (*Tlr3*, *Tlr4*, *Tlr7*, *Tlr8* and *Tlr9*), interleukins (*Il18* and *Il15*) and TNF pathway-related genes (*Tnfsf10*, *Tnfsf11* and *Tnfsf13b*) (Figure 3). A full list of the genes found in each category is presented in the interactive website http://bg.upf.edu/C10-HDAC7/ (for statistics see Dataset S1). To validate our microarray analysis, we selected several representative genes and tested their mRNA levels by RT-qPCR. We observed that the presence of HDAC7 significantly interfered with the increase in the mRNA levels of *Cxcl10*, *Fcgr1*, *Ifi35*, *Ifi47*, *Tlr3*, *Tlr9*, *Il18* and *C3* genes after β-estradiol treatment (Figure 4). Importantly, HDAC7 re-introduction did not interfere with the down-regulation of key genes for B cell differentiation and biology such as the transcription factor PAX5, reinforcing the notion that HDAC7 is a repressor of macrophage genes in B cell precursors (Figure S5). During the trasdifferentiation of C10 cells into macrophages the cells stop dividing and many genes related to the cell cycle, such as genes involved in mitosis, become down-regulated [17]. We have observed that the presence of HDAC7 also interferes with the down-regulation of these types of genes, corroborating that HDAC7 blocks, at least in part, the cellular transdifferentiation process (Figure S4, Dataset S2, and interactive website http://bg.upf.edu/C10-HDAC7/). Our data clearly demonstrate that HDAC7 represses the expression of central genes for macrophage function, and strongly suggest that HDAC7 acts as a specific transcriptional repressor of lineage inappropriate genes in B cell precursors.

### HDAC7 knock down leads to the de-repression of macrophage genes

**Figure 5:**
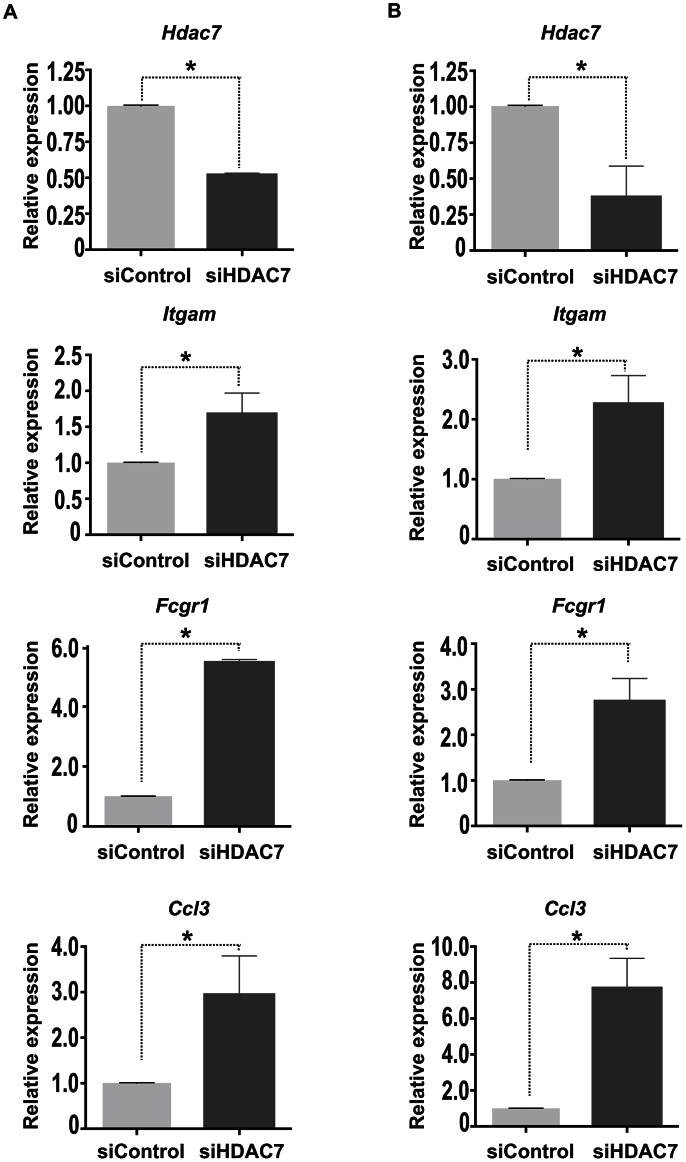
HDAC7 knock down leads to the de-repression of macrophage genes. HAFTL cells (A) and primary B cell precursors (B) were transfected with control siRNA or siRNAs targeting HDAC7. 72 hours after transfection *Hdac7*, *Itgam*, *Fcgr1* and *Ccl3* mRNA levels were determined by RT-qPCR experiments. Data are represented as the mean +/− standard error of the mean (SEM) of three independent experiments. The two-way ANOVA test was used to calculate significant levels between the indicated groups. *P<0.001.

To determine the physiological function of HDAC7 in B cell precursors and test whether it is involved in the repression of macrophage genes, we performed a loss of function experimental approach. We knocked down HDAC7 by siRNA in both HAFTL cells and primary pre-B cells (Figure 5). Strikingly, we observed that the reduction in HDAC7 mRNA levels resulted in the de-repression of key macrophage genes such as *Itgam* (Mac-1), *Fcgr1* and *Ccl3* (Figure 5). These data demonstrate that HDAC7 is involved in the repression of lineage inappropriate genes in B cell precursors.

### HDAC7 binds to the transcription factor MEF2C in pre-B cells and is recruited to the promoters of target genes critical for macrophage function

**Figure 6:**
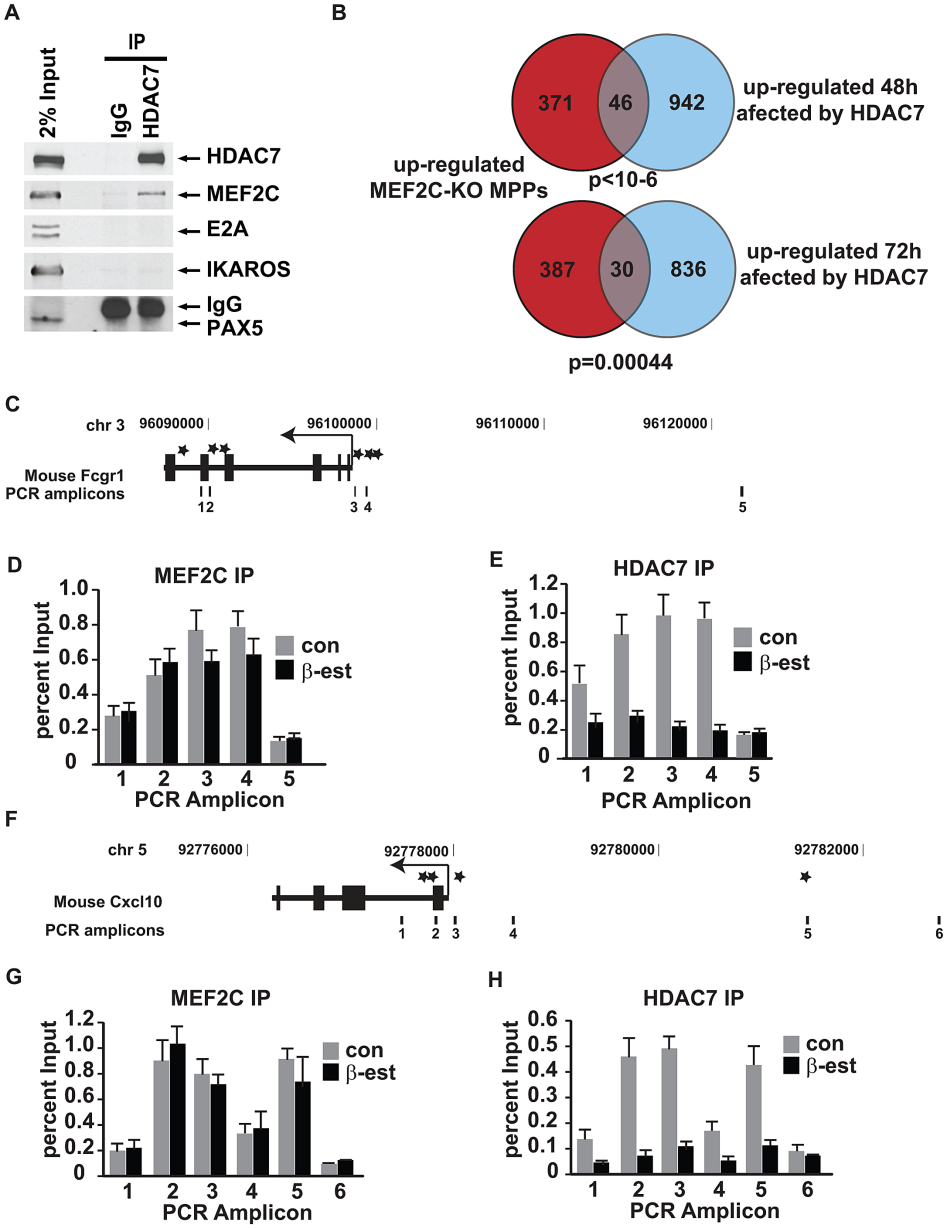
HDAC7 binds to the transcription factor MEF2C and is recruited to the promoter of key genes for macrophage function in pre-B cells. A. Co-immunoprecipitation experiments showing the specific binding of HDAC7 with MEF2C in pre-B cells. HDAC7 does not bind with IKAROS, PAX5 and E2A. B. Venn diagrams showing the total number of genes up-regulated in MEF2C deficient LMPPs, genes up-regulated at 48 and 72 hours of β-estradiol treatment affected by HDAC7 and the overlapping between both conditions. C and F. Schematic representation of the mouse *Cxcl10* and *Fcgr1* locus and amplicons scanned in Chromatin immunoprecipitation experiments by qPCR. Asterisks indicate MEF2 binding sites location. D, E, G and H. Chromatin immunoprecipitation experiments showing the enrichment of HDAC7 and MEF2C to putative MEF2 binding sites on the *Cxcl10* and *Fcgr1* gene loci in pre-B cells. Results are presented as percentage immunoprecipitated over input and are representative of three independent experiments.

At the mechanistic level, class IIa HDACs exert their actions as transcriptional repressors by interacting with specific transcription factors recruited to the promoters of genes required for development and cell differentiation [15], [16]. To address whether HDAC7 specifically interacts with particular sequence-specific transcription factors in pre-B cells, we undertook a candidate approach and performed co-immunoprecipitation experiments to test for potential interaction between endogenous HDAC7 and the B cell transcription factors IKAROS, E2A, PAX5, and MEF2C in the C10 parental line (HAFTL cells). HDAC7 specifically associated with MEF2C, but not with the other B cell transcription factors tested (Figure 6A). Recently, Camargo and colleagues have reported that MEF2C is crucial in the cellular choice towards the lymphoid versus the myeloid lineage in LMPPs [5]. Microarray analysis of control and MEF2C-deficient LMPPs revealed that the absence of MEF2C resulted in the up-regulation of genes enriched in GO categories related to the immune system, such as genes involved in the inflammatory and defense response of the cells [5]. Comparison of these data with our set of genes whose up-regulation was affected by HDAC7 re-expression during cellular transdifferentiation showed a significant overlap by Chi-square test of 46 (p<10^−6^) and 30 (p = 0.00044) genes, respectively (Figure 6B). Strikingly, the phagocytosis-related genes *Fcgr1*, *Fcgr2b* and *Fcgr3* were found to be targets of both HDAC7 and MEF2C. These data indicate that MEF2C represses genes characteristic of macrophages in hematopoietic progenitors and suggest that HDAC7 may also silence lineage inappropriate genes at earlier stages of lymphocyte development.

To test whether in pre-B cells HDAC7 is recruited to the promoters of macrophage-specific genes whose up-regulation is impaired in the presence of exogenously expressed HDAC7, we performed chromatin immunoprecipitation (ChIP) assays. Notably, using the TFconsite bioinformatic tool we found that promoters of *Fcgr1*, *Cxcl10* and *Itgam* contain putative MEF2 binding sites. We designed primers spanning the upstream regulatory regions, gene body, and downstream regulatory regions of mouse *Fcgr1*, *Cxcl10* and *Itgam* (Figure 6C and 6F and Figure S6A). Chromatin prepared from C10 cells un-induced or induced with β-estradiol for 72 hours was subjected to ChIP with antibodies specific for MEF2C and HDAC7. qPCR analysis of the immunoprecipitated material with primers specific for the *Fcgr1*, *Cxcl10* and *Itgam* loci indicated that both MEF2C and HDAC7 were specifically and significantly enriched at the identified putative MEF2 binding sites in un-induced C10 cells (Figure 6D, 6E, 6G and 6H and Figure S6B). Importantly, while significant MEF2C enrichment around its putative binding sites was found in both un-induced and induced conditions, HDAC7 enrichment at the target genes was lost upon β-estradiol induction (Figure 6D, 6E, 6G and 6H and Figure S6B). Taken together these data indicate that via interaction with MEF2C, HDAC7 is recruited to the promoters of myeloid genes in B cell precursors, resulting in their transcriptional silencing.

### HDAC7 re-expression interferes with the functional properties of the reprogrammed macrophages

**Figure 7:**
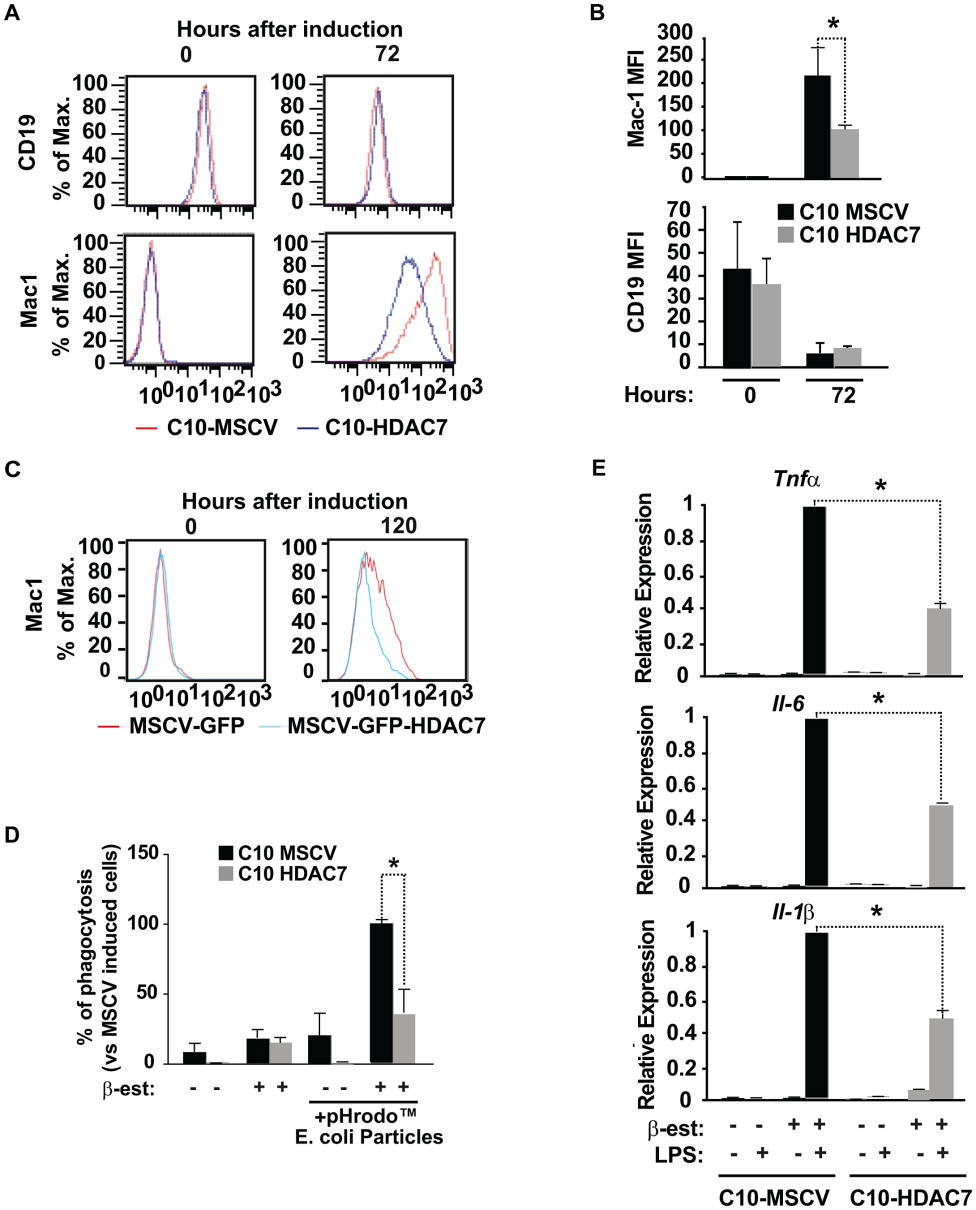
HDAC7 re-expression interferes with the functional properties of the converted macrophages. A. Histograms for Mac-1 and CD19 expression levels in C10-MSCV and C10-HDAC7 cells untreated or treated with β-estradiol for 72 hours. B. Mean fluorescence intensity (MFI) of Mac-1 and CD19 of C10-MSCV and C10-HDAC7 cells untreated or treated with β-estradiol for 72 hours. Data are represented as the mean +/− standard error of the mean (SEM) of three independent experiments. *P<0.001. C. Primary pre-B cells were transduced with the indicated retroviral vectors and 48 hours after induce to transdifferentiate. Histograms for Mac-1 proteins levels are shown. D. Capacity of C10-MSCV and C10-HDAC7 cells untreated or treated with β-estradiol for 48 hours to phagocytose red fluorescence bacteria. Data are given as mean ± SEM of values obtained in three independent experiments. Statistical significance was determined by two-way ANOVA followed by Bonferrony multiple comparison test. *P<0.001. E. Effect of HDAC7 expression in LPS-mediated *Tnfα*, *Il-1α* and *Il-6* gene expression. C10-MSCV and C10-HDAC7 cells were treated or not with β-estradiol for 48 hours. Then, the cells where incuated or not with LPS for 6 hours and RNAs analyzed by RT-qPCR. Data are represented as the mean +/− standard error of the mean (SEM) of three independent experiments. P values were calculated by the two-way ANOVA test. *P<0.001.

We previously reported that during reprogramming of pre-B cells into macrophages there is an increase in the expression of Mac-1 and a down-regulation of CD19 levels, two cell surface markers characteristic for macrophages pre-B cells, respectively. The reprogrammed macrophages show high phagocytic activity and respond to LPS treatment with cytokine production [17]. To test whether the presence of HDAC7 could interfere with the functional characteristics of the reprogrammed macrophages we undertook three different experimental approaches. First, we followed the expression kinetics of CD19 and Mac-1 (CD11b), two cell surface markers characteristic for pre-B cells and macrophages, respectively by flow cytometry. In both C10-MSCV and C10-HDAC7 cells, 100% of the population became CD19 negative and Mac-1 positive 72 hours after addition of β-estradiol (Figure 7A). However, the presence of exogenous HDAC7 resulted in a significant block in the expression levels of Mac-1 (Figure 7A and 7B). In contrast, CD19 protein levels decreased to the same extent regardless of the presence of HDAC7 (Figure 7A and 7B). Exogenous expression of HDAC7 in primary B cell precursors also interfered with the up-regulation of Mac-1 in transdifferentiation experiments (Figure 7C). These results demonstrated that in the presence of HDAC7 the reprogrammed cells are not able to express the levels of Mac-1 protein normally present in macrophages. Second, we tested the phagocytic properties of the transdifferentiated C10 cells exogenously expressing HDAC7. Interestingly, C10-HDAC7 cells treated with β-estradiol showed a significantly reduced capacity to phagocytose red fluorescence protein-expressing bacteria compared to the control C10-MSCV cells (Figure 7D). Lastly, we analyzed the inflammatory response of both cell lines after treatment with LPS in the presence or absence of β-estradiol for 48 hours. Strikingly, expression of HDAC7 resulted in a significant inhibition in the expression of the pro-inflammatory cytokines, *Tnfα*, *Il-1α* and *Il-6*, by the macrophage-like converted cells in response to LPS (Figure 7E). We next tested whether HDAC7 re-expression results in the reduction of Mac-1 protein levels once the cells have been reprogrammed into macrophages. We first treated C10 cells with β-estradiol for 72 hours and the reprogrammed macrophages were transduced with a retroviral vector carrying HDAC7-Flag. We observed a decrease in the expression levels of Mac-1 24 hours after expression of exogenous HDAC7 (Figure S7A). However, we did not observe any effect on either the expression of macrophage genes or the phagocytic capacity of RAW cells expressing exogenous HDAC7 (Figure S7B, S7C and S7D). We have recently shown that C/EBPα-mediated reprogramming of pre-B cells into macrophages occurs in the absence of significant changes in the DNA methylation of crucial genes suggesting that the reprogrammed macrophages retained an epigenetic memory characteristic of the cell of origin [19]. Therefore, we speculate that the chromatin structure present in differentiated macrophages is not permissive for the recruitment of HDAC7 to its target genes and that such recruitment is only possible in a chromatin environment characteristic of B cells, in line the notion that HDAC7 is a lymphoid-specific transcriptional repressor. Taken together, these results demonstrate that HDAC7 significantly interferes with key functional characteristics of the transdifferentiated macrophages.

### Interaction of HDAC7 with MEF2C and its catalytic activity are essential for repression of Mac-1

**Figure 8:**
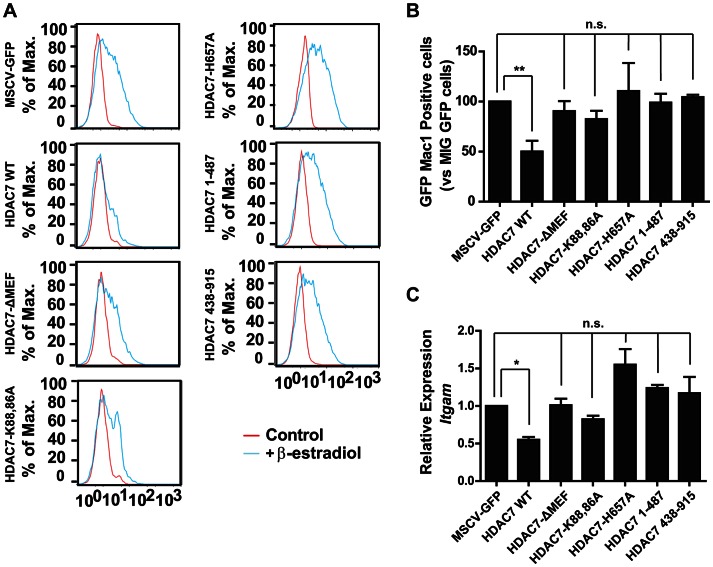
Interaction of HDAC7 with MEF2C and its catalytic activity are essential for repression of Mac-1. C11 cells were infected with MSCV-GFP, MSCV-GFP-HDAC7, MSCV-GFP-HDAC7(ΔMEF), MSCV-GFP-HDAC7(K86A/K88A), MSCV-GFP-HDAC7(H657A), MSCV-GFP-HDAC7(1–487) and MSCV-GFP-HDAC7(438–915) viruses and induced with β-estradiol for 48 hours. A. Cells were stained with a Mac-1 antibody and the GFP-positive fractions were gated and the results plotted. B. Percentage of Mac-1 positive cells treated with β-estradiol for 48 hours. Data are given as mean ± SEM of values obtained in three independent experiments. Statistical significance was determined by two-way ANOVA followed by Bonferrony multiple comparison test. *P<0.0001. C. RT-qPCR experiments for *Itgam* gene expression changes in cells treated with β-estradiol for 48 hours. Data are given as mean ± SEM of values obtained in three independent experiments. Statistical significance was determined by two-way ANOVA followed by Bonferrony multiple comparison test. *P<0.001.

Class IIa HDACs interact with MEF2 proteins via a conserved motif of 17 amino acids located in the amino-terminal region of the proteins. To definitively prove that HDAC7 represses macrophage genes through its interaction with MEF2C, we generated retroviral vectors carrying mutants of HDAC7 with either a deletion of the entire 17 amino acids stretch (HDAC7-ΔMEF) or with substitutions of crucial lysine residues (HDAC7-K86AK88A). We tested the HDAC7 mutants for their ability to repress Mac-1 during the transdifferentiation of pre-B cells into macrophages. Expression of wild-type HDAC7 resulted in a significant decrease in Mac-1 positive cells and mRNA levels (Figure 8) whereas expression of the HDAC7 mutants defective for MEF2C binding had no significant effect. We next tested whether the enzymatic activity of HDAC7 is necessary for its repressive action on Mac-1 expression during the conversion of pre-B cells into macrophages. We generated a retroviral vector for HDAC7 mutated in its catalytic domain (HDAC7-H657A), a C-terminal truncated construct HDAC7(1–487) that completely lacks the HDAC catalytic domain but contains the MEF2 interacting motif and a N-terminal truncated construct HDAC7(438–915) bearing the enzymatic motif but lacking the MEF2 domain. As shown in Figure 8, forced expression of wild-type HDAC7 interfered with the up-regulation of Mac-1 protein levels 48 hours after induction of cellular reprogramming. In contrast, the HDAC7-H657A, the HDAC7(1–487) and the HDAC7(438–915) constructs did not block the up-regulation of Mac-1 levels. These experiments demonstrate that both the HDAC7-MEF2C interaction, as well as its catalytic activity, are necessary for HDAC7 to repress macrophage genes during cellular reprogramming.

## Discussion

**Figure 9:**
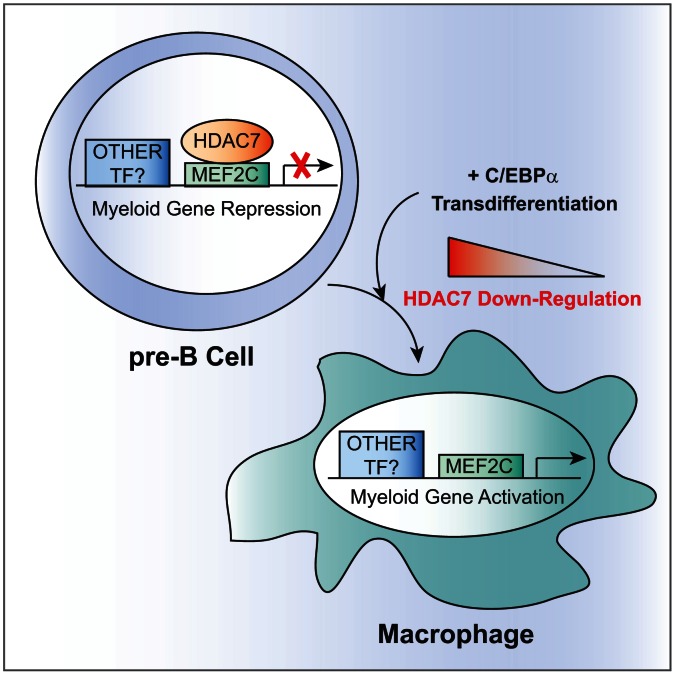
Model for HDAC7-mediated transcriptional repression in pre-B cells. HDAC7 is expressed in pre-B cells and not in macrophages. In pre-B cells, HDAC7 specifically interacts with the transcription factor MEF2C and is recruited to promoters of myeloid genes. During transdifferentiation of pre-B cells into macrophages HDAC7 is down-regulated allowing for expression of macrophages specific genes.

Our findings have revealed that HDAC7 is expressed in B cell precursors and not in macrophages (Figure 9). Using a cellular transdifferentiation system we have demonstrated that HDAC7 represses the expression of a large number of macrophage genes during the conversion of pre-B cells into this myeloid cell type. More importantly, depletion of HDAC7 in pre-B cells results in the de-repression of key macrophage genes. Functionally, the presence of HDAC7 interferes with the acquisition of key functional features of the converted macrophages. These aberrant macrophage-converted cells do not express the Mac-1 protein levels normally present in macrophages, are not able to properly phagocytose bacteria and do not respond adequately to endotoxin by expressing major pro-inflammatory cytokines. At the mechanistic level, HDAC7 specifically interacts with the transcription factor MEF2C in pre-B cells and is recruited to MEF2 binding sites located at the promoters of genes critical for macrophage function (Figure 6 and Figure S6). In addition to the interaction with MEF2C, the catalytic activity of HDAC7 is also necessary to repress macrophage genes. Our results demonstrate that HDAC7 is expressed in fetal and adult pre-B cells suggesting that it might play a role in both types of B lymphopoiesis.

Why is HDAC7 specifically expressed in pre-B cells and not macrophages? Based on our results, we conclude that HDAC7 is a key transcriptional repressor of lineage inappropriate genes in pre-B cells. Using a ChIP-seq approach Murre and colleagues have identified HDAC7 as a target of the transcription factors E2A, EBF and Foxo1 in B cell precursors (pro-B cells) postulating that it could be an important regulator of B cell development and indicating that HDAC7 function is not restricted to T cells within the hematopoietic system [21]. In addition, our results showing that in pre-B cells HDAC7 interacts with the transcription factor MEF2C, allowing the recruitment to the MEF2 binding sites on promoters of genes characteristic of macrophages reinforces our conclusion. Indeed, in the late nineties, MEF2C was found, among the different MEF2 family members, to be specifically expressed in B cells within the lymphocyte lineage, suggesting that it could have a role in B cell development and function [22]. More recently, it has been reported that MEF2C regulates B cell proliferation and survival after BCR activation and p38 MAPK signaling [23], [24]. MEF2C is also expressed at earlier stages of lymphocyte development [5], [25]. It has been reported that MEF2C is involved in the cellular choice towards the lymphoid versus the myeloid lineage in lymphoid-primed multipotent progenitors (LMPPs) [25]. At the molecular level MEF2C activates the transcription of lymphoid specific genes and represses myeloid genes [25]. We have observed a significant overlap between MEF2C regulated genes in LMPPs and our identified HDAC7 target genes in pre-B cells. Moreover, HDAC7 mutants that do not interact with MEF2C are unable to suppress the up-regulation of macrophage genes during the reprogramming process. Given this scenario, we propose that HDAC7 is the MEF2C transcriptional co-repressor responsible for the silencing of myeloid genes in B cells and lymphoid precursors.

Finally, in light of previous reports demonstrating the expression and functions of HDAC7 in T cells, our results have uncovered a more general role for HDAC7 in the lymphoid lineage compartment within the hematopoietic system. In addition, the finding that HDAC7 is dramatically down-regulated during the transdifferentiation of pre-B cells into macrophages not only reinforces this notion, but also provides an additional level of complexity to the way its activity and function are regulated. During T cell development in the thymus and later in a differentiated and specialized T cell type, cytotoxic T lymphocytes (CTLs), HDAC7 is regulated in a signal dependent manner responsible for its phosphorylation and for its subsequent nucleo-cytoplasmic distribution [26]–[31]. In resting thymocytes, HDAC7 is localized in the nucleus, where it represses the expression of a large number of genes involved in both positive (survival) and negative (apoptosis) selection of the cells. However, in response to TCR signaling, HDAC7 becomes phopshorylated and translocates to the cytoplasm where it can no longer repress its target genes [26], [27], [29], [31]. Later in the periphery, HDAC7 is constitutively phosphorylated and found in the cytoplasm of CTLs allowing the expression of key genes for the function of this specialized T cell type [30]. We could postulate that within a particular hematopoietic cellular lineage, the HDAC7 gene transcriptional repressive function is regulated in a signal-dependent way by controlling its phosphorylation status and altering its cellular distribution. That is, HDAC7 is unphosphorylated or phosphorylated and localized either in the cell nucleus or in the cytoplasm depending on the requirement to silence or activate specific genes at different stages of T cell development. In contrast, at the branching points where cellular lineage choice has to be made, e.g. lymphoid versus myeloid, HDAC7 might be regulated at the expression level. For instance, our results showing that HDAC7 expression is down-regulated during the conversion of pre-B cells into macrophages further support this hypothesis. However, as is the case for T cells, we cannot rule out the possibility that HDAC7 is additionally regulated in a signal dependent manner during B cell development and differentiation. In this regard, we have found that HDAC7 is expressed in the nucleus of pre-B cells (data not shown) probably due to the lack of a fully functional B cell receptor (BCR). However, it is highly probable that HDAC7 function is also regulated via BCR signaling at later cell differentiation stages when B cells develop into mature antibody-secreting cells.

Based on the findings of this study, we therefore propose that in the hematopoietic system, HDAC7 is not only a signal-dependent transcriptional repressor involved in different developmental steps of a particular lymphocyte type, but also a lineage-specific transcriptional repressor responsible for maintaining the identity of lymphocytes by silencing lineage inappropriate genes. We anticipate that other members of the class IIa HDACs subfamily might be specific repressors of undesirable genes in the cellular differentiation and developmental processes where they exert their actions (e.g. skeletal and cardiac muscle, bone formation and brain).

## Materials and Methods

### Plasmids

MSCV-puro-HDAC7, MSCV-GFP-HDAC7, MSCV-GFP-HDAC7(ΔMEF), MSCV-GFP-HDAC7(K86A/K88A), MSCV-GFP-HDAC7(H657A), MSCV-GFP-HDAC7(1–487) and MSCV-GFP-HDAC7(438–915) construct were generated by cloning the HDAC7-Flag cDNA PCR amplificated from the pCDNA3-HDAC7 wild-type and mutant plasmids previously described [20] into the MSCV-puro or MSCV-GFP vectors (Clontech).

### Antibodies

Anti-HDAC7 (H-273), anti-HDAC7 (C-18), anti-HDAC4 (H-92), anti-E2A (V-18), anti-PAX5 (C-20) and anti-RUNX1 (DW71) were purchased from Santa Cruz Biotechnology. Anti-IKAROS (ab26083) was purchased from Abcam; anti-MEF2C (D80C1) XP from Cell Signaling Technology; and anti-α-Tubulin (T61999), from Sigma-Aldrich.

### Cell culture and β-estradiol treatment

HAFTL cells were grown at 37°C in RPMI 1640 supplemented with 10% fetal bovine serum, 2 mM glutamine, and 50 U/ml streptomycin/penicillin. C10 cells (transduced with a MSCV-GFP-C/EBPα retroviral vector) and C11 cells (transduced with a MSCV-hCD4-C/EBPα retroviral vector) were cultured at 37°C in RPMI 1640 without phenol red supplemented with 10% of charcoal treated fetal bovine serum, 2 mM glutamine, and 50 U/ml streptomycin/penicillin. B-cell precursors and macrophages were obtained from mouse bone marrow as described [18]. For transdifferentiation induction, cells were treated with 100 nM β-estradiol in the presence of 10 nM of IL-3 and 10 nM of mCSF1 for the indicated periods of time.

### Retroviral supernatant generation and cellular transduction

For retrovirus generation the MSCV-puro, MSCV-puro-HDAC7, MSCV-GFP, MSCV-GFP-HDAC7, MSCV-GFP-HDAC7(ΔMEF), MSCV-GFP-HDAC7(K86A/K88A), MSCV-GFP-HDAC7(H657A), MSCV-GFP-HDAC7(1–487) and MSCV-GFP-HDAC7(438–915) plasmids were transfected into the packaged cell line Platinum-E and supernatant were collected at 48–72 hours post-transfection. For the generation of C10-MSCV and C10-HDAC7 cells, C10 cells were spin infected and 48 hours after were selected in the presence of 3 ug/ml of puromycin. C11 cells were spin infected with MSCV-GFP, MSCV-GFP-HDAC7, MSCV-GFP-HDAC7(ΔMEF), MSCV-GFP-HDAC7(K86A/K88A), MSCV-GFP-HDAC7(H657A), MSCV-GFP-HDAC7(1–487) and MSCV-GFP-HDAC7(438–915) and 48 hours after treated with β-estradiol.

### siRNA depletion

Dharmacon siRNA control and on-target smartpools targeting transcript of the mouse HDAC7 gene were used to knockdown its expression HAFTL cells and primary B cell precursors. siRNA were transfected using Lipofectamine RNAiMAX Transfection Reagent (Invitrogen). mRNA levels were examined by RT-qPCR experiments 72 hours after siRNA transfection.

### RT–qPCR experiments

RNA was extracted Trizol extraction (Qiagen) and cDNA synthesyzed using the High Capacity cDNA Reverse Trancription Kit (AB Applied Biosystems). RT-qPCR were performed in triplicate using SYBR Green I Master (Roche). PCR reactions were run and analyzed using the LightCycler 480 Detection System (Roche). Primers sequences upon request.

### Co-immunoprecipitation and Western blot analysis

Total cellular extracts were prepared in PLB buffer (0.5% Triton X-100, 0.5 mM EDTA, 1 mM DTT in PBS) supplemented with protease inhibitors (Complete, Roche Molecular Biochemicals). Immunoprecipitation, SDS-PAGE and Western blot experiments were performed as previously described [32].

### Flow cytometry

Cells were un-induced or induced to transdifferentiate. 48 hours later, cells were stained with fluorochrome conjugated antibodies against Mac-1 and CD19 (BD Pharmingen). Mac-1 and CD19 expression were monitored on a Gallios Flow Cytometer (Beckman Coulter) and analyzed by FlowJo software (Tree Star, Inc.).

### Phagocytosis assays

C10-MSCV and C10-HDAC7 cells were induced or not for 48 h and subjected to the pHrodo *E.coli* phagocytosis KiT (Invitrogen) following the manufacturers protocol.

### LPS induced inflammatory cytokines

C10-MSCV and C10-HDAC7 cells were induced to transdifferentiate. 48 h after induction they were incubated with LPS (1 µg/mL Sigma) for 6 h. RNA extraction, cDNA synthesis and RT-qPCR were performed as described above. Primers sequences upon request.

### Microarray experiments

Biological duplicates of C10-MSCV and C10-HDAC7 cells were un-induced or induced to transdifferentiate for 48 and 72 hours (12 samples in total). Total RNA from cultured cells was extracted by Trizol and then purified. PCR amplified RNAs were hybridized against Affymetrix mouse arrays chip (Mouse Genome 430 PM strip) at the IRB Genomics Facility. Affymetrix raw CEL files and processed (normalized) data have been deposited in GEO database under accession number GSE36827.

### Microarray data analysis

Affymetrix CEL files were background corrected, normalized using Bioconductor, package “affy” (version 1.28.1) using ‘expresso’ algorithm [33], [34]. Since the Affymetrix chip version used in this study contains only perfect match (pm) probes, for normalization and acquiring raw probe intensities to expression values we used the following parameters: background correction method “rma”; normalization method “constant”; pm correct method “pmonly”; and summary method “avgdiff”. Quality of microarray experiment (data not shown) was verified by Bioconductor package “arrayQualityMetrics” (version 3.2.4 under Bioconductor version 2.7; R version 2.12.1) [35]. To determine genes that are differentially expressed (DE) between two experimental conditions, Bioconductor package Limma was utilized to generate contrast matrices and fit the corresponding linear model [36]. Probe to gene annotation were performed using microarray vendor's annotation data. When more than one probe were annotated to same gene, highest absolute expression value was considered (maximizing). To consider a gene is differentially expressed, besides multiple test corrected, FDR p-value ≤0.05 as cut off, we also applied Log2 fold change (Log2FC) cut off 0.5 for β-estradiol treatment. We used Log2FC cut off 0.5 for genes that are affected by the expression of HDAC7 in β-estradiol treated cells. Expression data on Mef2c deficient multipotent progenitor cells were obtained from GEO database (accession No. GSE13686) [5]. Data were analyzed using the limma package from Bioconductor. Spots were not background corrected before within array loess normalization. After array normalization using the quantile method log2 ratios (mutant/control) was calculated. To define a gene up-regulated, we used Log2FC ≥1.0.

### Functional and pathway enrichment analysis

Functional annotation of differentially expressed genes is based on Gene Ontology (GO) (http://www.geneontology.org) as extracted from EnsEMBL and KEGG pathway database. Accordingly, all genes are classified into three ontology categories (i) biological process (BP), (ii) cellular component, (CC) and molecular function (MF) and pathways when possible. We have taken only the GO/pathway categories that have at least 10 genes annotated. We used Gitools for enrichment analysis and heatmap generation (www.gitools.org). Resulting p-values were adjusted for multiple testing using the Benjamin and Hochberg's method of False Discovery Rate (FDR).

### Chomatin immunoprecipitations assays

ChIP was performed essentially as previously described [32] on C10 cells un-induced or induced for 72 hours. To shear chromatin to an apparent length of ∼500 bp, chromatin was sonicated using a BioRuptor sonicator (Cosmo Bio Co., Ltd) with either 40 45-s pulses (uninduced cells) or 30 45-s pulses (induced cells) at maximum setting. Input and immunoprecipitated DNA were subjected to Sybergreen Q PCR cycles with specific primers (provided upon request).

## Supporting Information

Dataset S1 Microarray statistics analysis for genes up-regulated during the conversion of pre-B cells into macrophages that are affected by HDAC7.
(XLS)

Dataset S2 Microarray statistics analysis for genes down-regulated during the conversion of pre-B cells into macrophages that are affected by HDAC7.
(XLS)

Figure S1 HDAC7 is down-regulated during the transifferentiation of pre-B cells into macrophages. Kinetics of regulation (log2 Affymetrix expression values) of *Hdac7* and *Pax5* genes in C10 cells and primary pre-B cells transduced with a retroviral vector for inducible expression of C/EBPα. Cells were treated with β-estradiol for the times indicated. HAFTL cells, primary pre-B cells, primary bone marrow macrophages and RAW264.7 cells were used as control.
(EPS)

Figure S2 Western blot showing the protein levels of endogenous and exogenously expressed HDAC7 in C10 cells transduced with a untreated or treated with β-estradiol for 72 hours.
(EPS)

Figure S3 HDAC7 re-expression interferes with the gene transcriptional program of the converted macrophages. A. Heat-map statistics showing significantly (FDR p-value ≤0.05) enriched KEGG pathways among the up-regulated genes affected by the re-expression of HDAC7 during transdifferentiation of pre-B cells into macrophages. Colours toward red indicate high statistic significance, yellow indicates low statistic significance, and gray indicates no statistic significance. B. Heat-maps showing observed differentially expressed genes for selected KEGG pathways. Blue colour cell indicates positive events while gray colour indicates that the gene was not observed differentially expressed in that experimental condition.
(EPS)

Figure S4 HDAC7 re-expression interferes with the gene transcriptional program of the converted macrophages. Heatmap statistics showing significantly (FDR p-value ≤0.05) enriched A. GO Biological Processes categories, B. GO Cellular Components categories, C. GO Molecular Functions categories and D. KEGG pathways, among the down-regulated genes affected by the re-expression of HDAC7 during transdifferentiation of pre-B cells into macrophages. Colours toward red indicate high statistic significance, yellow indicates low statistic significance, and gray indicates no statistic significance.
(EPS)

Figure S5 HDAC7 re-expression does not interfere with the down-regulation of Pax5. A. Kinetics of down-regulation (log2 Affymetrix expression values) of *Pax5* in C10-MSCV and C10-HDAC7 cells un-treated or treated with β-estradiol for the times indicated. B. RT-qPCR validation of the results shown in A.
(EPS)

Figure S6 HDAC7 is recruited to the *Itgam* promoter in pre-B cells. A. Schematic representation of the mouse *Itgam* locus and amplicons scanned in Chromatin immunoprecipitation experiments by qPCR. Asterisks indicate MEF2 binding sites location. B. Chromatin immunoprecipitation experiments showing the enrichment of HDAC7 and MEF2C to putative MEF2 binding sites on the *Itgam* gene loci in pre-B cells. Results are presented as percentage immunoprecipitated over input and are representative of three independent experiments.
(EPS)

Figure S7 HDAC7 re-expression decreases Mac1 protein levels in the reprogrammed macrophages. A. Histograms for Mac-1 protein levels in reprogrammed machrophages transduced with either an empty vector or a retroviral vector for HDAC7 expression. B. Histograms for Mac-1 and CD19 protein levels in RAW-MSCV and RAW-HDAC7 cells. C. RT-qPCR experiments for gene expression changes for Hdac7, Itgam, Ccl3, and Fcgr1 genes in RAW-MSCV and RAW-HDAC7 cells. D. Capacity of RAW-MSCV and RAW-HDAC7 cells to phagocytose red fluorescence bacteria.
(EPS)
